# Cadaveric Evaluation of Injectate Distribution for Two Maxillary Nerve Block Techniques in Cats

**DOI:** 10.3389/fvets.2021.619244

**Published:** 2021-02-22

**Authors:** Lily V. Davis, Naomi K. Hoyer, Pedro Boscan, Sangeeta Rao, Jennifer E. Rawlinson

**Affiliations:** ^1^Colorado State University, Fort Collins, CO, United States; ^2^College of Veterinary Medicine and Biomedical Sciences, Colorado State University, Fort Collins, CO, United States; ^3^Epidemiology and Biostatistics, Department of Clinical Sciences, CVMBS, Colorado State University, Fort Collins, CO, United States

**Keywords:** maxillary nerve, cats, infraorbital approach, percutaneous approach, regional anesthesia

## Abstract

Regional nerve blocks have been shown to decrease general anesthetic drug requirements and improve pain management in patients undergoing surgery. Regional nerve blocks are used routinely in patients undergoing oral surgery, such as dental extractions. There is little published information regarding the efficacy of feline maxillary and infraorbital nerve blocks. The goal of the study was to compare injectate distributions of the infraorbital foramen and percutaneous maxillary nerve block techniques in feline cadavers using a combined dye and radiopaque contrast media solution to simulate an injection. There was no significant difference in length of stained nerve between the two different techniques. It was not necessary to advance the needle into the infraorbital canal to achieve effective staining of the maxillary nerve. There was no significant difference in injectate distribution between two different injectate volumes, 0.2 and 0.4 ml, indicating that the smaller volume injected at the infraorbital foramen resulted in adequate nerve staining.

## Introduction

A critical component of analgesia and anesthesia for feline patients undergoing maxillary dental and oral surgical procedures is the maxillary nerve block. The maxillary nerve block, when performed with chloroprocaine via the infraorbital technique, has been shown to reduce the reflex-evoked muscle action potential (REMP) associated with tooth-pulp stimulation of the maxillary teeth (1). It has also been shown that regional and local anesthetics decrease minimum alveolar concentrations (MAC) of gas anesthetics, and post-surgical pain in cats (2). Regional anesthetic drugs work by binding to voltage-gated sodium channels in their resting and inactive states preventing membrane depolarization, nerve excitation and conduction (3). When nerve blocks are performed accurately, action potentials are not propagated along the nerve, and the transmission of a nociceptive impulse is prevented (4). In order for a nerve block to be effective, a critical length of nerve must be circumferentially associated with an anesthetic drug. In myelinated nerves, this is approximately three nodes of Ranvier, which corresponds to ~6 mm of length in the frog sciatic nerves studied (5).

The maxillary nerves are branches of the sensory root of the trigeminal nerve (6). They provide sensory innervation to the maxillary teeth and soft tissue surrounding the orbit, maxillae, and snout. The maxillary nerve emerges from the rostral alar foramen into the pterygopalatine fossa and runs parallel with the maxillary artery on the surface of the medial pterygoid muscle. The infraorbital branch enters the maxillary foramen and passes through the infraorbital canal exiting via the infraorbital foramen. The caudal and middle superior alveolar branches exit the infraorbital nerve and the maxillary nerve in the pterygopalatine fossa in cats (6, 7). Inappropriately performed nerve blocks in cats have been associated with negative sequalae that include hematoma formation, and trauma to the targeted neurovascular structure and eye (8, 9). Several anatomical approaches for administering anesthetic drug to the maxillary nerves in cats have been described, and these include the maxillary tuberosity (or caudal intraoral), percutaneous (also known as the subzygomatic) and infraorbital foramen techniques (7, 10). When comparing the infraorbital foramen and percutaneous injection techniques in dogs, the infraorbital foramen technique had significantly greater maxillary nerve staining when a dye was used to simulate an injectate (11). Another similar study performed in cat cadavers, did not demonstrate the same difference (12).

This prospective observational study was designed to compare injectate distribution around the maxillary nerve branches between the infraorbital foramen and percutaneous injection techniques in cat cadavers. An additional aim of the study was to evaluate the injectate distribution of two injectate volumes, 0.2 and 0.4 ml. It was hypothesized that the infraorbital foramen technique and 0.4 ml injectate volume would provide superior nerve staining.

## Materials and Methods

The study design was reviewed and approved by the Colorado State University Clinical Trials Review Board, and due to the cadaveric nature of the study, no Institutional Animal Care and Use Committee protocol was necessary.

Sample size was determined by the number of cadaveric specimens available. Twenty cat cadavers of unknown age and body weight, euthanized at a separate facility for reasons other than this study, were provided for the study. Exclusion criteria included maxillofacial trauma, tumors changing osseous architecture of the bones of the skull, and deciduous dentition. Cadavers were stored in freezer quality bags at two degrees Fahrenheit, and heads were removed from the bodies at approximately the second cervical vertebrae with a high-speed band saw immediately prior to thawing. The heads were thawed in fresh bags at forty-four degrees Fahrenheit for 24 h prior to being utilized. After thawing, one head had to be excluded based on the presence of severe trauma. All heads were considered mesaticephalic. Computed tomographic (CT) imaging was obtained for all specimens on a 16 slice CT scanner^1^. Images were acquired in 2 mm transverse slices pre and post contrast injection and reconstructed into 2 mm standard and 1 mm bone algorithms. All CT images were acquired after positioning the heads with the mandible lowermost.

**Figure 1:**
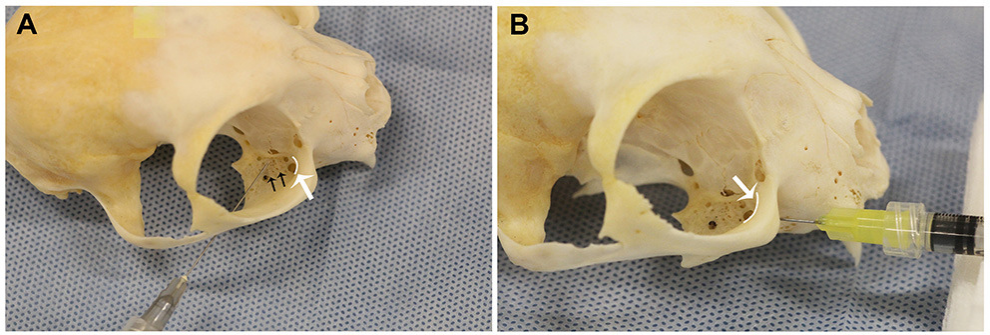
Needle position for the right **(A)** percutaneous (PC) and **(B)** infraorbital techniques, shown on a dry skull. Note that the needle does not exit the maxillary foramen in the IFR technique. The white curved lines, indicated by the white arrows, represent the maxillary foramen. The black arrows **(A)**, indicated superior alveolar foramina.

For the percutaneous technique (PC), 27G × 1.25” (0.4 × 32 mm) needles were used^2^. The technique was performed as previously described (10). The needle was inserted percutaneously between the rostroventral zygomatic arch and rostral border of the coronoid process of the mandible orienting medio-rostrally toward the estimated location of the maxillary foramen (Figure 1A). The needle was gently advanced until the tip of the needle engaged into the bone near the estimated location of the maxillary foramen. At this point, the needle was pulled back ~2–3 mm to the location of the superior alveolar branches of the maxillary nerve, at which point the injection was performed.

For the infraorbital foramen technique (IFR), 27G × 0.5” (0.4 × 13 mm) needles were used (Cite to Foot Note 2). The technique was performed as previously described, with one modification (10). The infraorbital foramen was identified intraorally via palpation dorsal to the maxillary third premolar tooth. The needle was inserted through the oral mucosa into the infraorbital foramen only (Figure 1B). Unlike previously described techniques, the needle was not advanced into the infraorbital canal. The injectate was deposited, and after removal of the needle, digital pressure was applied over the infraorbital foramen for ~10 s.

The PC and IFR techniques were performed on each of the 19 cadaver specimens randomly assigning which technique was performed on the right vs. left side and alternating which technique was performed first. In addition, two different volumes of injectate were compared, 0.2 and 0.4 ml. Because of the exclusion of one specimen, 0.4 ml of injectate was used on 10 specimens, 0.2 ml of injectate was used on nine. For the initial group of 10 specimens, each side was injected with both 0.2 ml iohexol (240 mg/ml) and 0.2 ml New Methylene Blue Dye for a total volume of 0.4 ml per site ^3^,^4^. The contrast and dye were mixed in the same syringe for both injections. The injections were done using 1 ml tuberculin syringes ^5^. The injections were performed on the right side of the specimen, then the left side of the specimen. The CT was obtained after both injections were performed. The 10 specimens were injected and imaged together as a group. Three weeks later, in a second set of 9 specimens, the volume of the dye and contrast injectate was decreased to 0.2 ml total volume (0.1 ml dye and 0.1 ml iohexol). This group of nine heads were injected and scanned together. All injections were performed by the same investigator (NH), positioning the cadavers in sternal recumbency. CT images was acquired pre- and immediately post-injection. Post-injection CT images were qualitatively reviewed to screen for the presence of contrast material within the eye and determine the location of the injectate within the infraorbital canal.

When imaging was complete, the eyes were dissected out of the head using a previously described enucleation technique (13). The region of dye staining was identified and measured within the ventral orbit. Dye on the ventral exterior portion of the globe and extraocular tissues was recorded. Dye distribution was measured in millimeters using both calipers and a UNC-15 periodontal probe^6^,^7^. The probe was utilized within the orbit because the calipers would not fit into that location. After dye distribution was recorded, the maxillary nerve was dissected from the rostral alar to the infraorbital foramen. The nerve was identified as it entered the pterygopalatine fossa and severed with a 15 blade at that site. The oral mucosa was elevated in the region of the infraorbital foramen and the exposed infraorbital nerve was severed as it exited the foramen. The nerve was dissected away from the floor of the orbit and measured on a flat surface with calipers.

The data were checked for a normal distribution. A Fisher's exact test was used to determine statistical significance. A *p*-value of 0.05 was used to determine statistical significance. The same statistical software was used for all statistical analyses^8^. Means and standard deviations were calculated for all parameters and compared across the results. Comparison of injectate distribution and dye staining of the nerve between the techniques were analyzed using a chi-square analysis.

## Results

**Figure 2:**
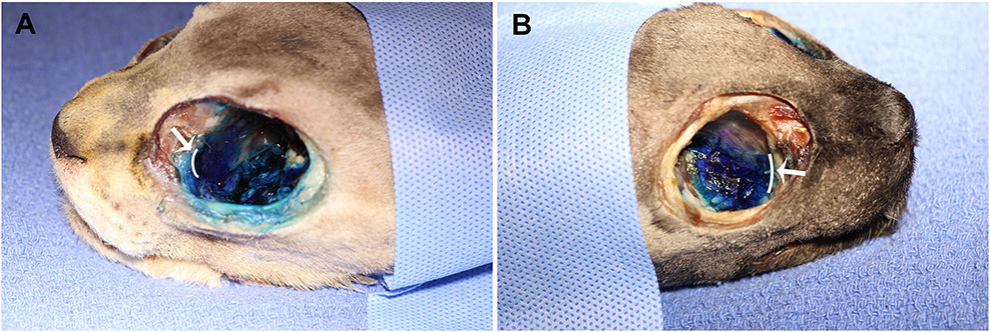
Dye distribution with 0.4 ml volume with the **(A)** PC and **(B)** IFR techniques in two of the cadaver specimens. The white curved lines, indicated by the white arrows, represent the maxillary foramen.

**Figure 3:**
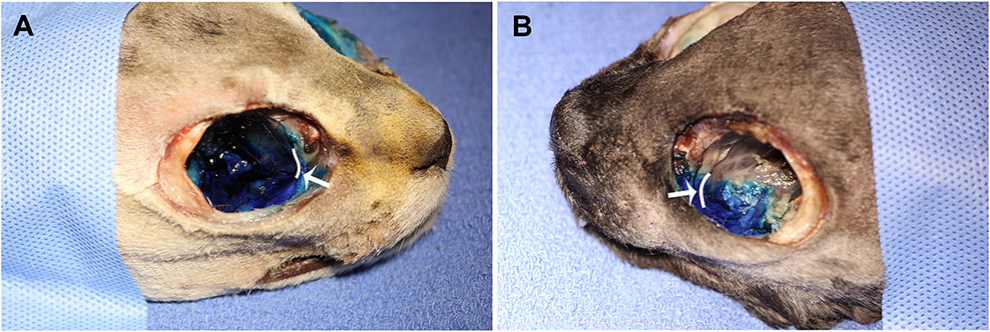
Dye distribution with 0.2 ml volume with the **(A)** PC and **(B)** IFR techniques in two of the cadaver specimens. The white curved lines, indicated by the white arrows, represent the maxillary foramen.

**Figure 4:**
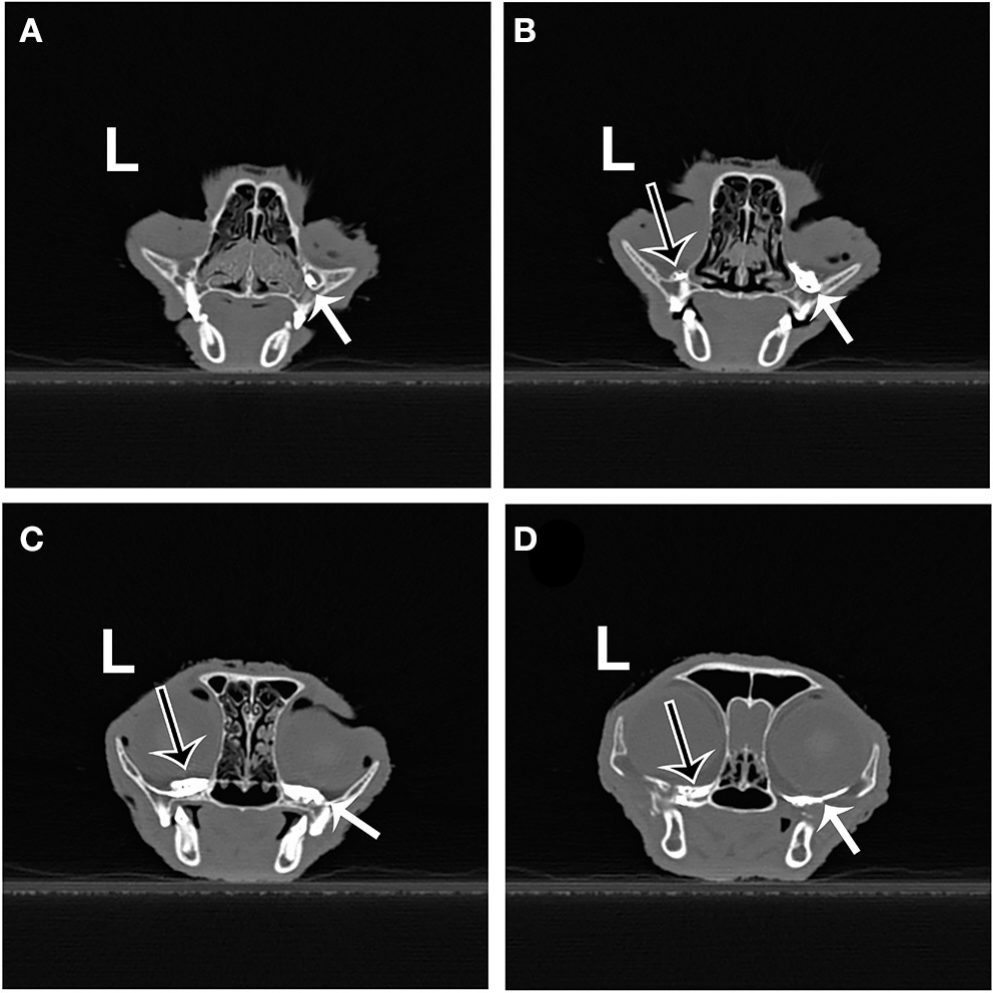
Transverse CT images from the same cadaver specimen after injection of 0.2 ml of contrast using the IFR (right side) and PC (left side) techniques. **(A)** Contrast material (white arrow) present in the right infraorbital canal (IFR technique). **(B)** Contrast material on the left (black arrow outlined in white) and right (white arrow) orbital floor, just caudal to the maxillary foramen. **(C,D)** Contrast material present further caudal into the suborbital region on both sides.

No contrast was identified within the globe with CT for either injection technique or volume. Table 1 indicates the number of specimens associated with ≥6 mm of dye staining for both injection methods and volumes of injectate. There was no significant difference between both injection techniques and volumes of injectate regarding circumferentially stained nerve lengths. Figures 2, 3 show typical dye distribution for both the PC and IFR techniques. There was no significant difference between injection techniques regarding injectate distribution along the floor of the orbit or ventral globe. For the larger injectate volume, the range for length of nerve associated with circumferential dye staining was 15–35 mm with a mean of 24 mm (std ± 6 mm), and for the smaller injectate volume the range was 26–44 mm with a mean of 34 mm (std ± 5 mm). For the IFR technique, the dye and contrast were present at the infraorbital foramen, and then coursed distally. For the PC technique, the dye was present in the ventral orbit in all but one injection (97.4%). One injection with the PC technique led to no stained coverage of the nerve due to inadvertent injection under the palatal mucosa. The needle was inadvertently placed ventral to the hard palate. For both volumes of injectate, only the IFR technique led to contrast being visualized within the infraorbital canal on CT imaging (Figure 4).

**Table 1:** Number of maxillary nerve samples associated with dye ≥6 mm, measured in millimeters, after percutaneous (PC) and infraorbital (IFR) injection techniques, using 0.2 and 0.4 ml of injectate volume.

**Approach**	**Injected volume (ml)**	**Number of injected heads (n = )**	**Maxillary nerve staining (≥6 mm)**
IFR	0.2	9	9
	0.4	10	10
PC	0.2	9	8*
	0.4	10	10

*One out of nine injections was erroneously performed*.

## Discussion

The hypothesis that the IFR technique and difference in injectate volume would provide superior nerve staining and injectate placement was rejected. No significant difference in either injectate distribution was found when comparing injection technique and volume of injectate. However, in one of the PC injections the injectate missed the targeted area completely, and there was no dye staining associated with the nerve. Interestingly, these findings are different than those found by Becerra et al. (12). In the Becerra study, a catheter was used to perform the IFR technique. This technique was not repeated in this study because the authors wished to reproduce the technique most commonly used in their clinical cases. Becerra's conclusion was that the IFR approach was not recommended for maxillary nerve block in cats. However, all the investigators in that study performing the blocks were previously untrained in the IFR technique. This may indicate that adequate training in the technique is important for success.

Previous descriptions of how to perform the IFR technique involve catheter placement into the infraorbital canal (10, 12). The length of the infraorbital canal in feline mesaticephalic skulls is roughly 4–5 mm: therefore, a catheter was deemed unnecessary for this study. In addition, even though the needle was not advanced into the infraorbital canal with the IFR technique, the maxillary nerve was associated with adequate dye staining, indicating that placement of the needle at the infraorbital foramen will provide adequate nerve coverage. This is relevant clinically, because trauma to the eye has been previously reported with the IFR technique (9).

It was surprising that there was no statistically significant difference in stained nerve lengths when comparing the two different injectate volumes. Even small volume of anesthetic circumferentially stained a mean of 33.8 mm of nerve, which is suggestive that even smaller volumes of injectate may be able to achieve the 6 mm minimum for effective loss of nociceptive nerve transmission.

A very recent study performed in canine cadavers demonstrated that injectate distribution after injection of contrast into the peribulbar compartment led to the intra-cranial presence of injectate in all cases where peribulbar injection was achieved (14). While distribution caudally from the pterygopalatine fossa was not evaluated in this study, the ability of injectate to distribute through the alar foramina intra-cranially is another reason to minimize injectate volume. This study did not evaluate possible sequelae of intra-cranial injection of regional anesthetic agents.

One factor that is impossible to convey in the data reported is the variability in the fat present in the ventral floor of the orbit. It is possible that variable amounts of periocular fat could impact the effectiveness of the PC technique when administered in live patients. In the PC technique, the needle is advanced deep into the orbital region, in the direction of the maxillary foramen, but it is not possible to visualize the precise location of the advanced needle. Due to the nature of the injection, it is critical that the needle used for this technique be as thin and as short as possible to avoid damage to adjacent structures.

There are several limitations associated with this study design. The small number of cadaver specimens and the lack of brachycephalic specimens made it impossible to evaluate the effect of the anatomic variability on the accuracy of the injections. The fact that the cadavers were frozen prior to the study may have altered the tissue and caused abnormalities in injectate distribution. Distribution of injectate in cadaveric specimens may not be reflective of distribution in live cats due to different tissue physical properties and active circulation. An additional limitation was the lack of randomization in the injected amounts of dye. All the injections with 0.4 ml were performed in one group of cadavers, while the injections of 0.2 ml volumes were performed several weeks later. It is possible that some learning occurred from one group to another, improving accuracy. In addition, the simulated injectate does not match the density and fluidity of typical regional anesthetic drugs. It would have been ideal to utilize CT imaging to evaluate needle placement to ensure that the injectate was being delivered accurately. However, the current study more closely mimics the techniques that would be performed in actual patients. One final limitation was the lack of turgor associated with the globe of the eye. This may have artificially reduced the risk of globe penetration compared to a live cat.

Finally, it warrants pointing out that even though 6 mm of nerve staining should be adequate for providing effective regional anesthesia, injectate distribution and nerve staining does not equate to maxillary nerve block effectiveness. Additional studies are needed to compare the effectiveness of the two techniques in providing regional anesthesia in a live patient. Given the findings that every specimen had >6 mm of dye staining and dye was present on the ventral eye of all the samples, a future study is warranted to evaluate even lower injectate volumes. In conclusion, 0.2 ml of injectate delivered via the PC and IFR techniques proved to be effective in achieving acceptable circumferential nerve staining of the maxillary nerve in cat cadavers.

## Data Availability Statement

The original contributions presented in the study are included in the article/supplementary material, further inquiries can be directed to the corresponding author/s.

## Ethics Statement

Ethical review and approval was not required for the animal study because this study was evaluated by the Clinical Review Board of our institution and it was determined that an IACUC review was not needed because of the cadaveric nature of the research.

## Author Contributions

LD worked with the NH to obtain funding and complete the project between her 2nd and 3rd years of veterinary school. PB was the advising anesthesiologist for the paper. SR assisted LD with the statistical analysis. JR and NH worked together with LD to generate the idea for the project. JR was also instrumental in providing editing feedback. All authors contributed to the article and approved the submitted version.

## Conflict of Interest

The authors declare that the research was conducted in the absence of any commercial or financial relationships that could be construed as a potential conflict of interest.

## Footnotes

^1^Philips Gemini TF BigBore System, Philips Medical Systems, Cleveland, OH.

^2^Covidien Monoject Standard Hypodermic Needle, Medtronic, Minneapolis, MN.

^3^Omnipaque iohexol solution, Novaplus, Winnetka, IL.

^4^Compounded at Colorado State University's Veterinary Teaching Hospital, Fort Collins, CO.

^5^Covidien, Medtronic, Minneapolis, MN.

^6^6-inch plastic medical calipers, Alibaba Group Hangzhou, China.

^7^UNC-15 periodontal probe, Shipps Dental, and Specialty Products Marana AZ.

^8^SAS v9.4, SAS Institute Inc., Cary, NC.
